# Jaw-specific differential kinase activity profiles in human periodontal ligament stem cells under mechanical compression

**DOI:** 10.1186/s13287-025-04778-5

**Published:** 2025-11-07

**Authors:** Christian Niederau, Sanne L. Maas, Emiel P. C. van der Vorst, Leon J. Schurgers, Yang Shi, Frank Hölzle, Michael Wolf, Rogerio B. Craveiro

**Affiliations:** 1 Department of Orthodontics, University Hospital RWTH Aachen, Pauwelsstraße 30, 52074 Aachen, Germany; 2 Department of Internal Medicine I, Aachen-Maastricht Institute for Cardio-Renal Disease (AMICARE), Institute for Molecular Cardiovascular Research (IMCAR), University Hospital Aachen, RWTH Aachen University, Aachen, Germany; 3 Institute for Cardiovascular Prevention (IPEK), Ludwig-Maximilians-Universität, Munich, Germany; 4 Department of Biochemistry, CARIM, Maastricht University, Maastricht, The Netherlands; 5 Institute of Experimental Medicine and Systems Biology, RWTH Aachen University, Aachen, Germany; 6 Department of Polymer Therapeutics, Institute for Experimental Molecular Imaging, RWTH Aachen University Clinic, Aachen, Germany; 7 Department of Oral and Maxillofacial Surgery, University Hospital RWTH Aachen, Pauwelsstraße 30, 52074 Aachen, Germany

**Keywords:** Upper jaw, Lower jaw, Kinase activity, Kinomics, PamGene, Human PDL, PDLSC, hPdlF, Tooth movement, Maxilla, Mandible

## Abstract

**Background:**

Increasing clinical and experimental evidence suggests that the upper and lower jaw exhibit pronounced functional and structural disparities that cannot be explained solely by classical anatomical differences. A central, yet unresolved question is whether periodontal ligament stem cells (PDLSCs) from these distinct regions display site-specific molecular signaling patterns in response to mechanical stress. We propose that PDLSCs derived from maxillary and mandibular regions activate distinct kinase signaling networks upon mechanical stimulation, thereby establishing a jaw-specific mechanobiological fingerprint.

**Methods:**

PDLSCs were isolated from seven healthy donor teeth and exposed to a static compressive force of 2 g/cm^2^. Kinase activity was profiled using a high-throughput PamChip^®^ peptide arrays that target both protein tyrosine kinases (PTKs) and serine/threonine kinases (STKs). Downstream pathway enrichment analyses were conducted using the Wikipathways, Gene Ontology (GO), KEGG, and Enriched pathways databases.

**Results:**

Mechanical stimulation induced distinct kinase activation signatures depending on jaw origin. Maxillary PDLSCs displayed a predominance of STK activation, while mandibular cells showed a relative reduction in PTK signaling. Only two PTKs and four STKs were consistently regulated across both regions, supporting the presence of a region-specific mechanotransduction profile.

**Conclusion:**

These findings support our hypothesis that localized differences in kinase signaling may constitute a molecular basis for the clinically observed jaw-specific phenomena, such as heterogeneous orthodontic tooth movement and alveolar bone remodeling. Jaw-dependent mechanotransduction pathways can therefore be considered key determinants of periodontal biology and may provide a basis for follow-up studies aimed at enabling the development of personalized orthodontic and regenerative strategies.

**Supplementary Information:**

The online version contains supplementary material available at 10.1186/s13287-025-04778-5.

## Introduction

The periodontium, a multifunctional tissue complex, serves as the principal anchoring system for teeth within the alveolar bone, integrating mechanical support with biological responsiveness. It consists of mineralized and soft tissues, including the alveolar bone, periodontal ligament (PDL), cementum, and gingiva, which work in concert to maintain stability and respond to mechanical forces [1, 2]. The most common cells within the PDL are periodontal ligament stem cells (PDLSC). As a central component of the periodontal apparatus, they are key regulators of the microenvironment and essential for tooth attachment and nutrition, as well as performing functions of proprioception, buffering of acting forces, enabling periodontal remodelling, and transmission of mechanical signals. The extracellular matrix produced by PDLSCs constitutes the structural framework surrounding the tooth root and plays a pivotal role in maintaining tissue homeostasis within the periodontium [3–6]. 

When orthodontic forces are applied, the tooth moves within its bony socket, resulting in the formation of zones of tension and compression [7, 8]. Mechanical stress applied to the PDL initiates a cascade of biological processes that enable controlled tooth movement (OTM) through the bone. Here, the PDL plays a central role by transmitting mechanical forces through collagen fibers, particularly Sharpey’s fibers, while maintaining flexibility [9, 10]. 

Intercellular crosstalk within the periodontal microenvironment is essential in orchestrating localized tissue remodeling and preserving the functional integrity of the periodontium [1]. Mechanical stress-induced remodeling of soft and hard periodontal tissue involves a coordinated interplay among osteoclasts, osteoblasts, fibroblasts, vascular networks, and immune cell populations within the PDL [8, 9]. 

In compression zones, osteoclasts, which reach the local area from periodontal blood vessels [11, 12], can resorb the alveolar bone to enable tooth movement in the direction of the applied force, while in tension zones, osteoblasts deposit new bone to stabilize the tooth. This dynamic remodelling is regulated by multiple signalling pathways that control the release of RANKL/OPG and cytokines amongst others, which are essential for bone remodelling processes by increased osteoclast activation [13]. 

These processes appear to differ between the upper (maxilla) and lower jaws (mandible), which have different anatomical and physiological features that impact wound healing, disease prevalence, and the speed of orthodontic tooth movement. For example, orthodontic tooth movement is significantly faster in the maxilla than in the mandible [14–16]. This difference is primarily due to the structural composition of the bones. The upper jaw consists mainly of trabecular bone, which is less dense and more porous, allowing for faster bone remodelling during OTM. In contrast, the mandible contains a higher proportion of cortical bone, which is denser and more resistant to resorption and deposition processes [17]. This structural disparity slows down the rate at which teeth can move in the mandible compared to the maxilla [18, 19]. Additionally, vascularization plays a significant role in these upper and lower jaw-specific differences. The maxilla’s richer blood supply enhances cellular activity and may accelerate bone turnover, facilitating faster tooth displacement. Contrarary, the mandible’s relatively lower vascularization may contribute to slower remodeling [20, 21]. These findings underscore the necessity of incorporating jaw-specific biological characteristics into orthodontic treatment to enhance therapeutic precision and predictability.

Additionally, all these jaw-specific characteristics have been observed in functional clinical practice. A more detailed investigation into the molecular factors that promote jaw-specific clinical characteristics is needed. Only a few pioneering studies have investigated differences in cell signaling between human periodontal ligament stem cells from the upper and lower jaw under mechanical stimulation [22–24]. Most previous studies on periodontal remodelling and orthodontic tooth movement have not taken into account the jaw-specific differences between the upper and lower jaw. However, there are initial indications that the cytokine content in the gingival crevicular fluid in the upper jaw may differ from that in the lower jaw [23]. 

In a previous study without mechanical stimulation, we demonstrated that PDLSCs from the upper region exhibit significantly higher proliferation and differentiation potential than those from the lower region [6]. Further, under physiological conditions the kinase activity in the lower jaw appeared higher than in the upper jaw [25]. Based on these findings, we hypothesised that kinomics, which measures kinase activity, should reflect the key inherent features of PDLSC cell-specific regulatory networks from the maxilla and mandible that determine the regenerative potential of PDLSC and thus explain differences in clinical observations.

We propose that periodontal ligament stem cells derived from the maxilla and mandible activate distinct kinase signaling networks in response to mechanical stimulation, thereby establishing a region-specific mechanobiological fingerprint. To elucidate these signatures, we used PamChip^®^ peptide array-based assays to systematically profile kinase activity and downstream signaling pathways in human PDLSCs isolated from the maxilla and mandible of the same donor under standardized compressive force. These assays targeted phosphotyrosine kinases (PTKs) and phospho-serine/threonine kinases (STKs). This experimental strategy enables region-specific kinase activity signatures to be discriminated in genetically matched cells, minimizing donor variability and allowing subtle, jaw-dependent differences in mechanotransduction to be detected. By delineating these molecular signatures, we aim to identify the key regulatory kinases involved in mechanically induced periodontal remodeling, as well as the associated signaling pathways. These insights are expected to provide a conceptual framework for future pathway-centric research into the molecular dynamics of PDLSCs and could ultimately inform precision approaches in orthodontic and regenerative therapies, which are tailored to specific biological contexts.

## Materials and methods

### Study design

Human periodontal ligament stem cells were obtained from seven patients (4 male, 3 female, aged 18–31 years). Detailed information about the donors and the specific teeth is shown in Table 1. The collection and use of periodontal cells derived from discarded patient biomaterial was authorized by the Ethics Committee of the University of Aachen, Germany (approval number EK 374/19), and all procedures adhered to relevant ethical guidelines and regulations. Informed consent was secured from all participants or their legal representatives. Primary human PDLSCs were isolated and analyzed for the expression of specific stem cell markers as well as their differentiation into adipocytes, chondrocytes, and osteoblasts, as outlined in previous studies [6, 25]. Subsequently, human PDLSCs were cultured in six-well plates until reaching 90–100% confluence. After a 24-hour incubation period, a static compressive force of 2 g/cm² (0.02 N/cm²) was applied for 24 h to the monolayer using sterile round glass cylinders (34 mm diameter; 18 g), following the protocol established by Kanzaki et al. [26]

**Fig. 1:**
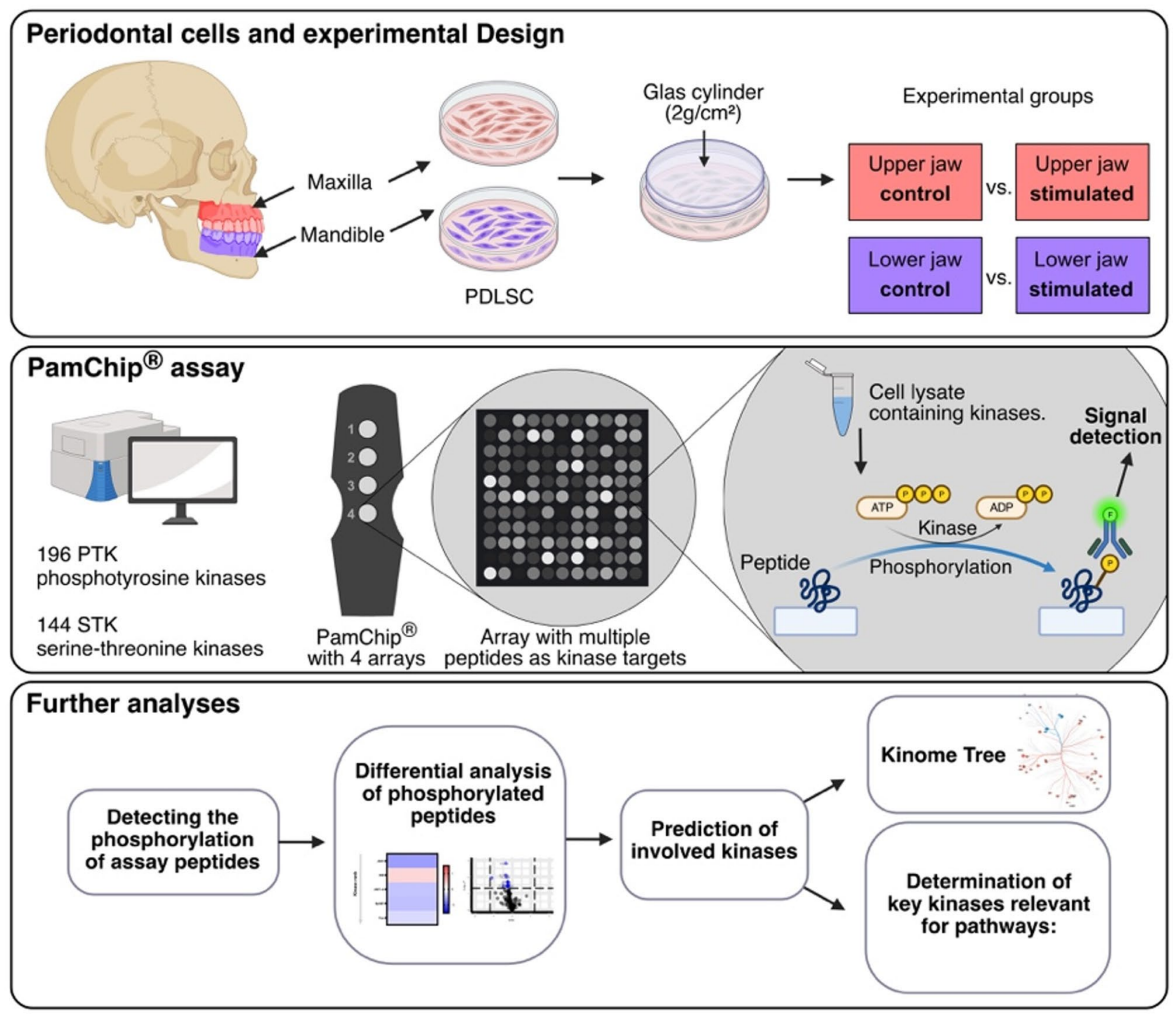
Schematic diagram of the study design. Molars from healthy donors were extracted from the upper and lower jaws of the same donors. The periodontal ligament (PDL) was removed from the tooth and the human periodontal ligament stem cells (hPDLSCs) were isolated. After an incubation period of 24 h, a static force of 2 g/cm² was applied to the monolayer using sterile round glass cylinders. The lysed cells were analysed using PamChips^®^ for PTK (tyrosine kinase) and STK (serine/threonine kinase) kinomics technology and kinomics analysis was performed

To obtain sufficient material, cells from two wells of a 6-well plate were lysed and combined into a single pooled sample for further processing. Total protein was quantified before loading the lysates onto corresponding PamChips^®^ for PTK (tyrosine kinase) and STK (serine/threonine kinase) analysis. This leads to 7 biological replicates from different donors analyzed in independent arrays on the PamChips^®^. Kinase activity profiles of PDLSCs derived from the upper and lower jaw of the same individual were then comparatively analyzed under conditions with and without applied compressive force (Fig. 1). Peptide phosphorylation patterns are represented in volcano plots, and kinase activity is visualized in heatmaps. Pathway analysis was performed with a primary focus on signaling pathways related to inflammation, hypoxia, vascularization, immune modulation, bone remodeling, and wound healing.

## Samples and cell culture

Human PDLSCs were obtained from the periodontal connective tissue of the middle root section of decay-free human teeth, freshly extracted for medical purposes at the dental clinic of the University Hospital RWTH Aachen. The isolation procedure followed previously published protocols [5, 24]. Isolated PDLSCs have previously been characterized based on criteria established by the International Society for Cellular Therapy, including the evaluation of specific surface markers and their ability to differentiate into adipocytes, chondrocytes, and osteoblasts in vitro [5, 24, 25]. 

## Tyrosine and serine/theonine kinase activity profilig

The PamChip^®^ microarray system on the PamStation^®^12 platform (PamGene International, s’Hertogenbosch, The Netherlands) was employed to analyze PTK and STK activity profiles. The PTK-PamChip^®^ arrays contain 196 unique phospho-sites, while the STK-PamChip^®^ arrays include 144 unique phospho-sites, representing peptide sequences derived from PTK and STK substrates, respectively. Phosphorylation of peptides was detected via fluorescence resulting from the binding of a FITC-conjugated antibody, which specifically recognizes the phosphorylated phospho-sites (Fig. 1).

Human PDLSCs from the maxilla and mandible were cultured in six-well plates at passage two until reaching approximately 80% confluence. Cells were washed with ice-cold PBS and lysed on ice for 15 min using M-PER Mammalian Extraction Buffer (Thermo Fisher Scientific, Waltham, USA) supplemented with Halt Phosphatase Inhibitor and EDTA-free Halt Protease Inhibitor Cocktail (1:100 each; Thermo Scientific). Lysates were centrifuged at 12,000 g for 15 min at 4 °C in a pre-cooled centrifuge, and the supernatant was collected. Protein concentrations were determined using the Pierce™ Coomassie Plus (Bradford) Assay (Thermo Fisher Scientific, Waltham, USA), according to the manufacturer’s protocol.

For the PTK assay, 10 µg of protein per array was used following the standard PamGene protocol with provided reagents. The PTK Basic Mix was prepared by combining freshly frozen lysate with 4 µL of 10× protein PTK reaction buffer (PK), 0.4 µL of 100× bovine serum albumin (BSA), 0.4 µL of 1 M dithiothreitol (DTT), 4 µL of 10× PTK additive, 4 µL of 4 mM ATP, and 0.6 µL of monoclonal FITC-conjugated anti-phosphotyrosine detection antibody (clone PY20). The final volume was adjusted to 40 µL with distilled water. Prior to loading the PTK Basic Mix onto the arrays, a blocking step was performed using 30 µL of 2% BSA followed by washing with PTK solution for preprocessing. Subsequently, 40 µL of PTK Basic Mix was applied to each array, and microarray testing was conducted over 94 cycles. Images were captured using a CCD camera on the PamStation^®^12 during kinetic read cycles 32–93 at exposure times of 10, 50, and 200 ms, as well as during end-level read cycles at exposure times of 10, 20, 50, 100, and 200 ms.

For the STK assay, each array was loaded with 2.0 µg of protein and 400 µM ATP along with an STK Basic Mix, consisting of 1× protein PTK reaction buffer (PK), 1× bovine serum albumin (BSA) and 1x STK antibody mix. Prior to loading the STK Basic Mix onto the arrays, a blocking step was performed using 30 µL of 2% BSA followed by washing with PTK solution for preprocessing. Subsequently, 40 µL of STK Basic Mix was applied to each array. A FITC-conjugated antibody was applied to visualize phosphorylation after one hour of incubation at 30 °C. During this incubation, samples were pumped back and forth through the porous material to enhance binding kinetics and reduce assay duration. Imaging was performed using an LED-based imaging device, and spot intensities were measured at each time point after adjusting for local background.

Data analysis was conducted using BioNavigator software version 6.3 (PamGene International). No blinding was performed in our study because all analyses were conducted using the standardized and fully automated PamChip^®^ assay platform, which relies on predefined, objective read-out parameters. Since proper balancing of the chips is required to ensure this standardisation, the operator must be aware of the conditions. The data acquisition and quantification are software-based and independent of operator interpretation, thereby minimizing the risk of observer bias.

## Prediction of upstream kinases

For both PTK and STK assay, Upstream Kinase Analysis (UKA), a functional scoring method (PamGene) was used to rank kinases based on combined specificity scores (based on peptides linked to a kinase, derived from 6 databases) and sensitivity scores (based on treatment-control differences). Data is represented as mean kinase statistics (adjusted p-value < 0.05) and node size median final score (>1.2). The specificity score reflects the association of a kinase with its corresponding peptides, as curated from six databases, while the sensitivity score captures the magnitude of phosphorylation changes between treatment and control conditions. The resulting median final score integrates these two components, providing a measure of the kinase’s overall activity. A median final score greater than 1.2 indicates a consistent and biologically relevant regulation of kinase activity across multiple peptides, distinguishing true activation events from background variation. Kinases meeting this threshold (adjusted p-value < 0.05) were considered significantly regulated and are represented in the network visualization by increased node sizes. For detailed information about the algorithms used, please refer to the PamChip^®^ documentation. The distribution of the phosphorylated peptides is shown in the volcano plots created with the R package EnhancedVolcano [27]. Blue dots depict peptides with an adjusted P value < 0.05. The active kinases are represented in a heatmap; the color scale indicates mean kinase statistic, and the kinase ranking is based on the median final score. Additionally, the kinases can be depicted in a coral tree to visualize the kinase families; here, node/branch colors represent mean kinase statistics, and node sizes represent the median final scores.

## Pathway analysis and differential analysis

Over-representation analysis (ORA) of the Kyoto Encyclopedia of Genes and Genomes (KEGG) database for the kinases with significant differences (median final scores >1.2 and adjusted p-value < 0.05) was performed using the R packages clusterProfiler [28] Data was visualized with the R package ggplot2 [29]. 

Pathway enrichment analysis was performed on the differentially expressed kinases. The R package disease ontology semantic and enrichment analysis (DOSE) [30] was utilized to analyze the biological complexities in which these kinases correlate with multiple annotation categories, which was visualized with the R package ggplot2 [29] in a dotplot and/or in a network plot with the help of the R package Reactome Pathway Analysis (ReactomePA) [31]. 

### Sample size calculation

The required sample size for the cell culture experiments was estimated a priori using a power analysis based on previously published results for the phosphorylation of pP38 [5]. The calculation was performed using GPower 3.1 [32], assuming an expected effect size of 2.453, a significance level (α) of 0.05], and a statistical power (1–β) of 0.95. Based on these parameters, a minimum of 6 independent samples per group was required to detect a statistically significant difference.

To ensure reproducibility and account for potential data loss or experimental variation, we included 7 independent biological replicates per condition in all experiments.

### Statistical analysis

Overall, the kinase data is represented as significantly different when the median final scores are higher than 1.2 and the adjusted p-value is lower than 0.05. All statistical analysis was conducted using R and are described in greater detail in the sections “Prediction of Upstream Kinases” and “Pathway Analysis and Differential Analysis” above.

## Results

The data presented on PDLSCs are based on self-isolated human PDLSCs from the upper and lower jaws of different donors and have already been characterized in our previous studies. The PDLSCs also showed similar characteristics in the evaluation of stem cell character [5, 6, 24, 25] as described in the basic protocols defined by the International Society for Cellular Therapy for mesenchymal stem/stromal cells and for periodontal ligament stem cells [33].

To determine protein kinase activity profiles of PDLSCs from the upper and lower jaw without stimulation and under compressive force, we applied PamChip^®^ technology (PamGene, Hertogenbosch, Netherlands) to analyze kinase activity in PDLSCs from seven donors.

### Phosphorylation of PTK-peptides in the lower jaw was more widely relugated than STK under compressive forces

Protein tyrosine kinase (PTK) analysis revealed that 137 PTK-specific peptides in the upper and lower jaw were phosphorylated. Of these, 11.67% (16/137) were significantly down-regulated in the lower jaw, whereas in the upper jaw 0.45% (3/137) were regulated (2 up-regulated and 1 down-regulated). STK-specific peptides showed that 87 peptides were phosphorylated in both jaws. Only up-regulated phosphorylation of peptides was observed in both jaws: 0.034% (3/87) in the lower jaw and 0.057% (5/87) in the upper jaw. Interestingly, only one peptide (RBL2-655-667) shows a significant change in phosphorylation in both the lower and upper jaw. All other significant differences are specific to each jaw. A detailed list of all peptides whose phosphorylation status changed significantly in response to compressive force stimulation in the upper and lower jaw can be found in Table 2 and Figs. 3A and 4A.

As a next step, Upstream Kinase Analysis was performed to identify the kinases that are potentially responsible for the phosphorylation patterns observed on PamChip^®^ peptides. The identified kinases were scored according to their statistical relevance to the corresponding peptide sets.

Following the prediction, an analysis of the upper jaw using PTK revealed that 73 kinases were active. Of these, 93.1% (68/73) were not significantly differently regulated. IRR (Insulin Receptor kinase) is the only up-regulated kinase in PDLSCs of the upper jaw under compressive force, compared to control, and accounted for 1.4% (1/73) of all kinases. In comparison, 5.5% (4/73, JAK3, JAK1b, ErhB3, and Fyn) of the kinases were down-regulated in these samples. PTKs from the lower jaw were found to be more down-regulated in PDLSCs after subjecting them to compressive force compared to control (57.5%; 42/73).

**Fig. 2:**
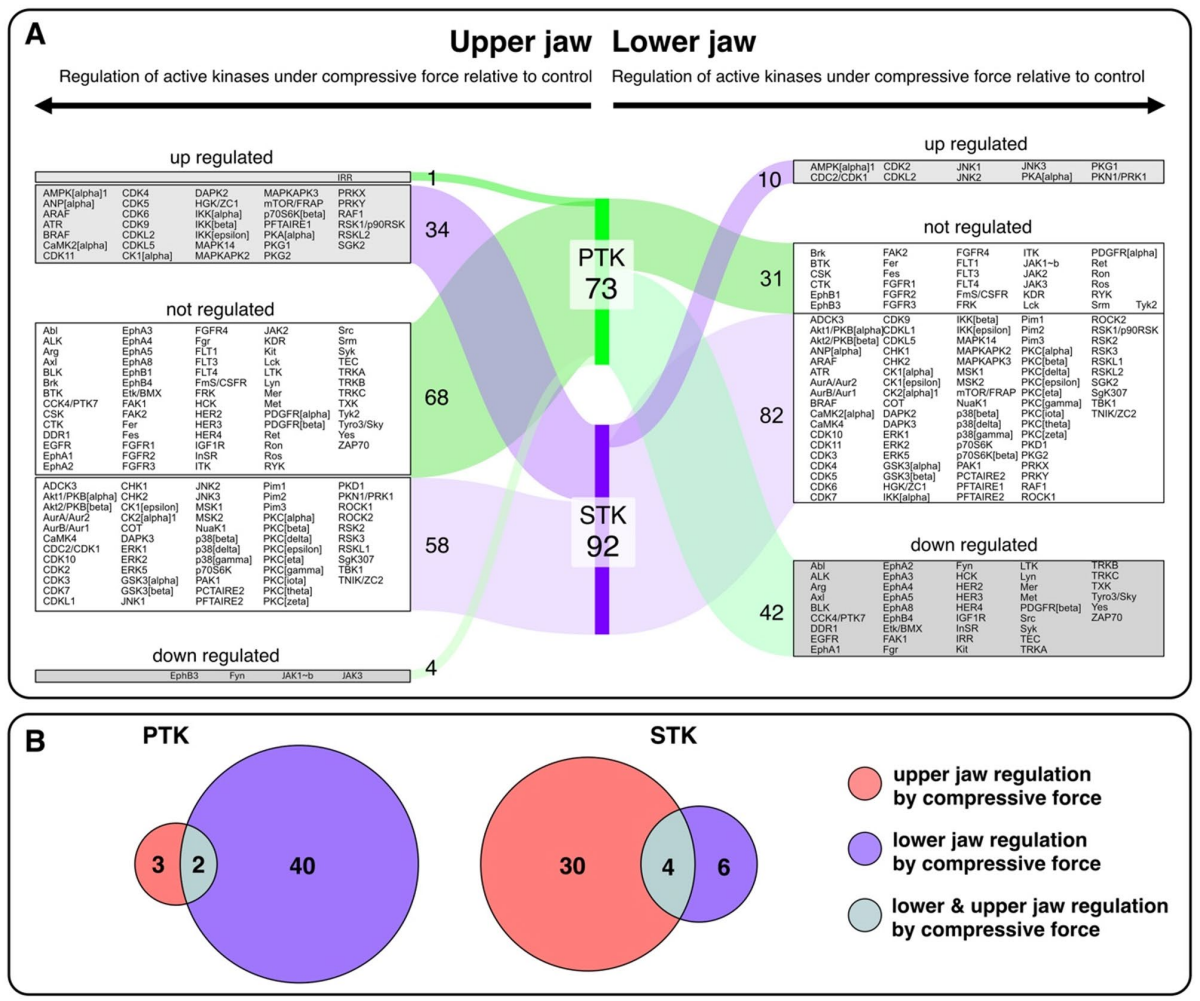
Regulation of active kinases. **A** Sankey diagram illustrating differential kinase activity profiles in periodontal ligament stem cells from the upper and lower jaw under compressive force compared to control. A pronounced difference in kinase activity was observed between PTK (tyrosine kinases) and STK (serine/threonine kinases) when comparing PDLSC derived from the maxilla (upper jaw) and mandible (lower jaw) under mechanical compression. The diagram visualizes shifts in phosphorylation patterns, highlighting distinct jaw-specific signaling responses. Not active kinases are not shown (123 PTK and 52 STK) **B** Comparison of regulated kinases between upper and lower jaw. (The data shown are derived from experiments performed with PDLSCs obtained from seven different donors)

Predicted STKs from the upper jaw showed that 92 kinases were active in PDLSCs exposed to force. In this case, 36.9% (34/92) were up-regulated, while 63% (58/92) were not significantly differently regulated. STKs from the lower jaw were less differently active compared to control: 10% (10/92) were up-regulated, while 90% (82/92) were not differently regulated between the two conditions. (Fig. 2). Table 3 provides a detailed listing of the top 10 regulated PTKs and STKs in PDLSCs under compressive force relative to control from lower and upper jaw.

### STK activity was strongly up-regulated and PTK activity showed slight differences under compressive force in the upper jaw

**Fig. 3:**
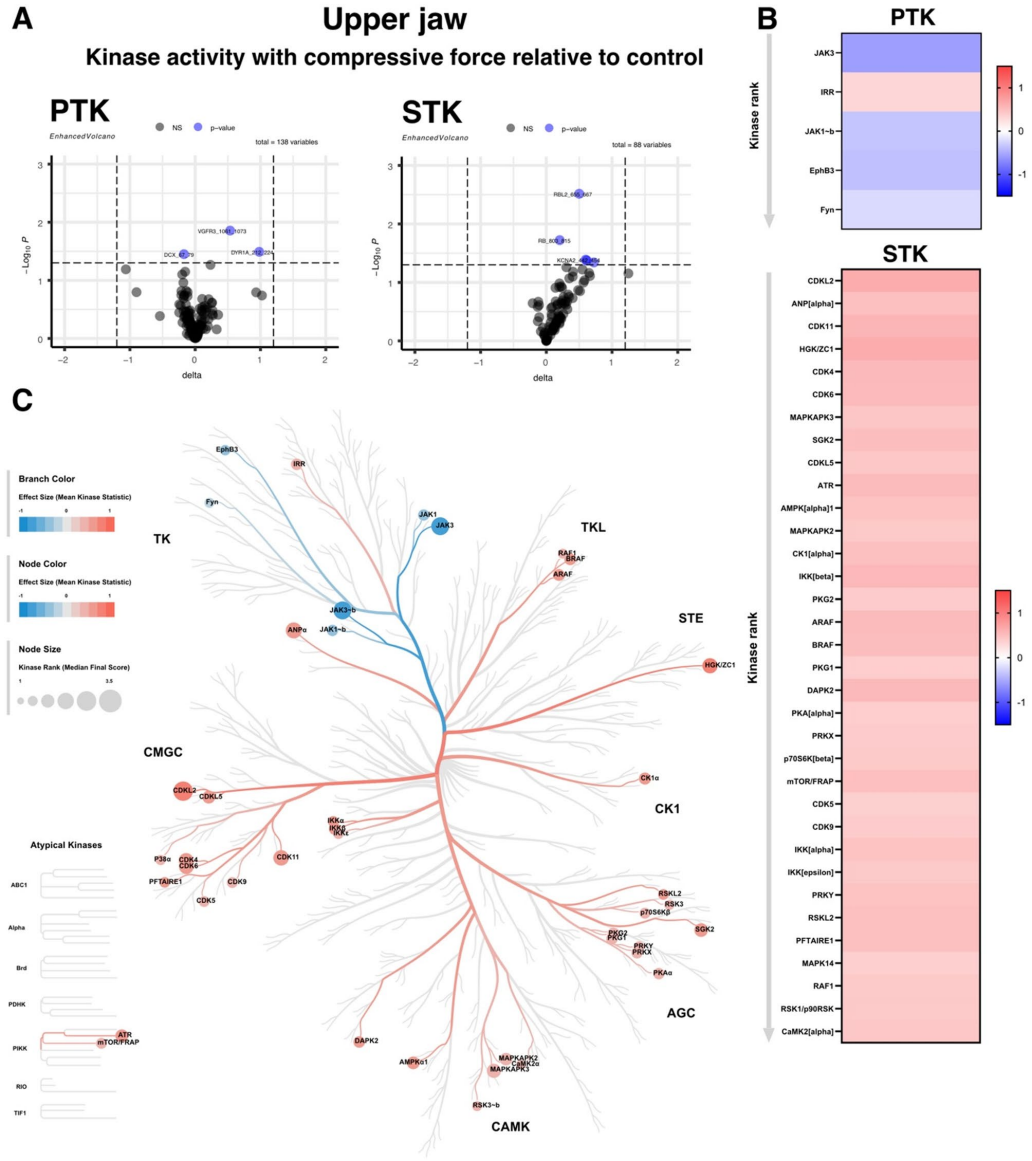
Compressive force induced a strong up-regulation of PTK phosphorylation in upper jaw. **A** Volcano plots of differentially phosphorylated PTK and STK peptides in arrays exposed to mandibular PDLSC lysates. The x-axis shows the effect size (delta) and the y-axis shows the significance (-Log10 p-value). Blue spots represent peptides whose phosphorylation levels are significantly different between the stimulated and control condition **B** Heatmap of significantly different kinase activity in upper jaw PDLSC, compressive force relative to control, without stimulation of seven donors. The red colour reflects increased activity, while the blue colour reflects decreased activity. **C** Combined PTK and STK kinome tree. Kinase activity is shown on the phylogenetic tree of the human protein kinase family. Dot size indicates specificity score and colour indicates kinase statistic (comparative force vs. control). PTK = phosphotyrosine kinase, STK = serine-threonine kinase. (The data shown are derived from experiments performed with PDLSCs obtained from seven different donors.)

The activity of STKs in the upper jaw was significantly up-regulated, while on the other hand, only a few PTKs were down-regulated. The kinases that had a significantly different activity in the upper jaw under compressive force compared to unstimulated control were visualized on the Coral kinome tree plot (Fig. 3C). The mean kinase statistics values (adjusted p-value, shown in Table 3), encoded in branch color, indicate the overall change of the peptide set that represents the kinase, with an adjusted p-value of < 0.05 indicating kinase upregulation of PDLSC from compressive force compared to control (Fig. 3B and C). Specific enhancement of STK was observed in a broad spectrum of kinase families of the human kinome. The CMGC kinase family, named after the initials of its main kinase families (CDKs, MAPKs, GSKs, and CLKs), includes key kinases: the MAPK growth- and stress-response kinases, the cell cycle (cyclin dependent kinases - CKD), and kinases involved in splicing and metabolic control. This kinase family regulates the progression through the different phases of the cell cycle in association with their activating partners, cyclins. The calcium and calmodulin-dependent protein kinase family (CAMK) is involved in the phosphorylation of transcription factors and, therefore, in the regulation of expression of responding genes. The casein kinase family (CK1) is also involved in cell cycle, transcription and translation, the structure of the cytoskeleton, cell-cell adhesion and receptor-coupled signal transduction and protein kinase A, G, and C family (AGC) (Fig. 3C).

### PTK activity was strongly down-regulated under compressive force in the lower jaw

**Fig. 4:**
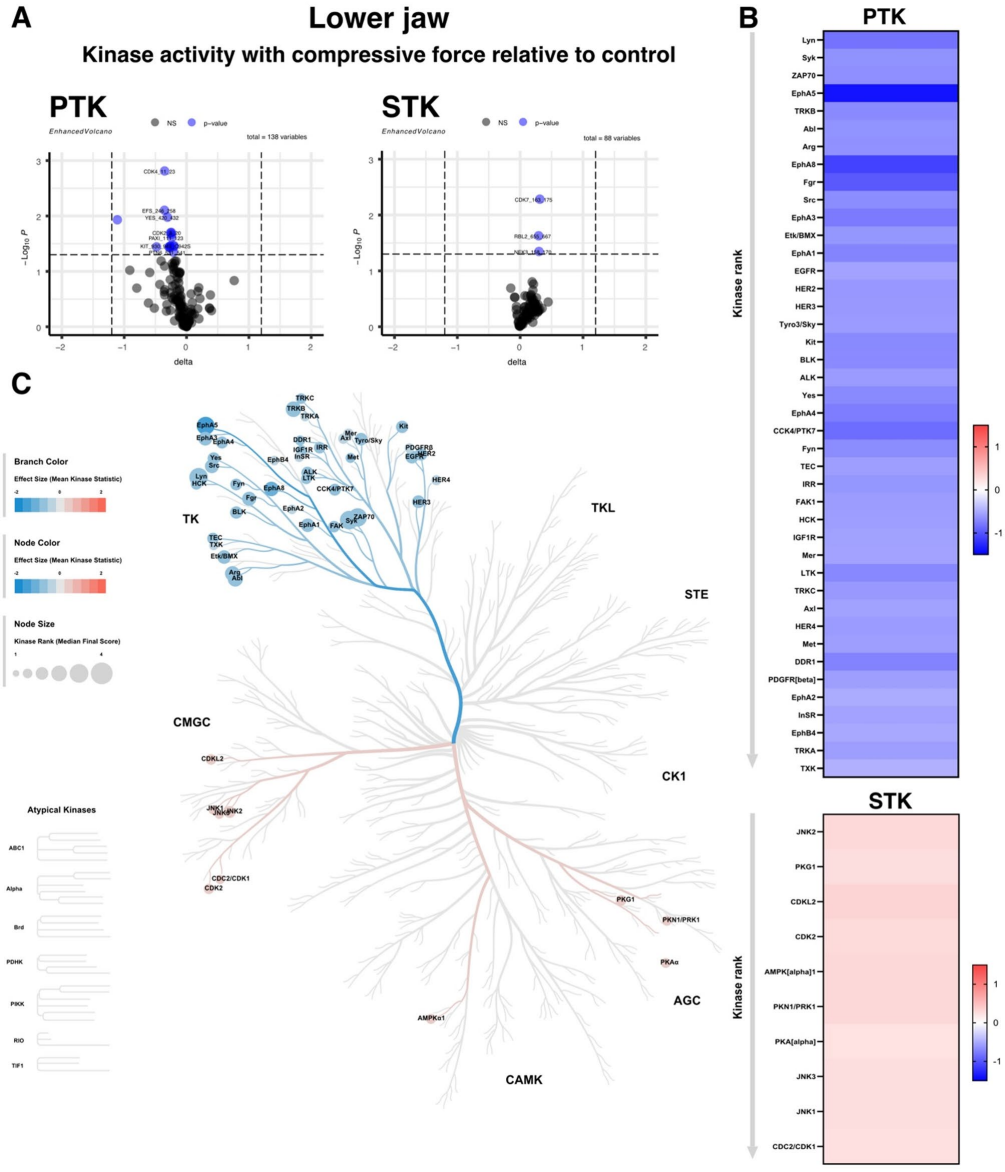
Compressive force induced a strong downregulation of PTK activity in lower jaw. **A** Volcano plots of differentially phosphorylated PTK and STK phosphosites in arrays exposed to lower jaw PDLSC lysates. The x-axis shows the effect size (delta) and the y-axis shows the significance (-Log10 p-value). Blue spots are peptides whose phosphorylation is significantly different between the stimulated and control condition (*p* < 0.05, unpaired t-test). **B** Heatmap of significant differently kinases activity in lower jaw PDLSC, compressive force relative to control. Red colour reflects increased activity, while blue colour reflects decreased activity. **C** Combined PTK and STK kinome tree. Kinase activity is shown on the phylogenetic tree of the human protein kinase family. Dot size indicates specificity score and colour indicates kinase statistic (comparative force vs. control). PTK = phosphotyrosine kinase, STK = serine-threonine kinase. (The data shown are derived from experiments performed with PDLSCs obtained from seven different donors.)

In contrast to the upper jaw, the lower jaw exhibited a strong downregulation of the tyrosine kinase (TK) family (Fig. 4). This kinase family plays a crucial role in transmitting extracellular signals into the cell: more than half of all TKs are receptor tyrosine kinases (RTKs) located at the cell surface. At the same time, many of the remaining members function near the cell membrane. Additionally, the lower jaw showed upregulation of STK activity, specifically involving kinases from the CMGC and AGC families, as well as AMPKα from the CAMK family.

### Selection of signalling pathways revealed different regulatory network analysis in the maxilla and mandible under compressive forces

**Fig. 5:**
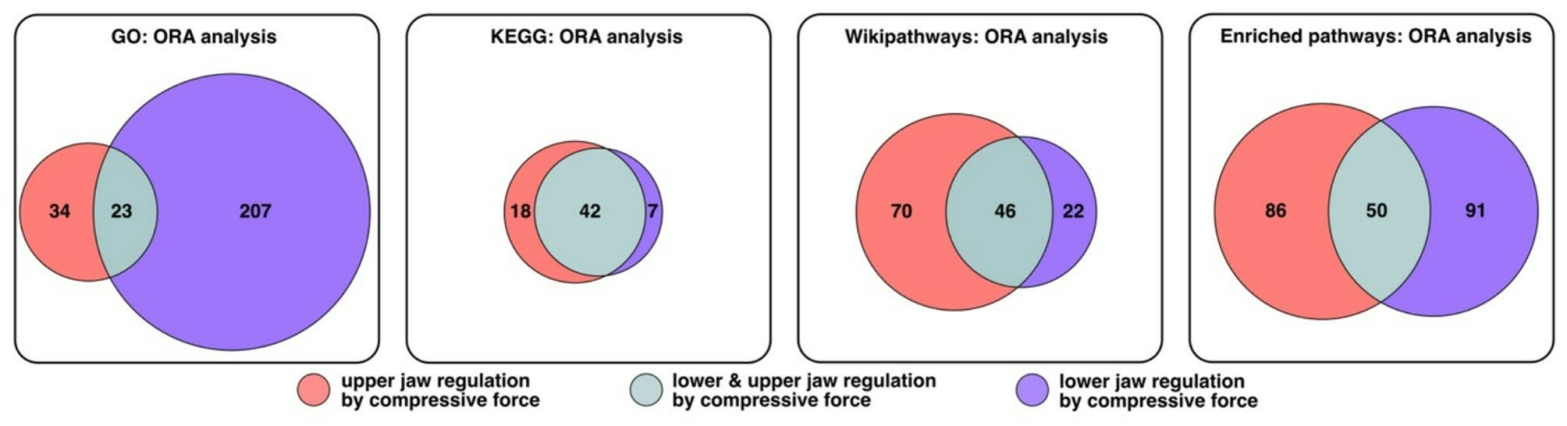
Overrepresentation analysis (ORA)

In order to gain mechanistic insights into the regulatory pathways underlying the differential development of the maxilla and mandible under compressive forces, we performed ORA to the most important processes in the periodontal remodelling of periodontal ligament cells under compressive forces. For this purpose, four data bases were included, namely the Gene Ontology (GO), Kyoto Encyclopedia of Genes and Genomes (KEGG), Wikipathways and Enriched pathways. Table 4 shows the selected signalling pathways that potentially contribute to key biological processes in the remodelling of the periodontium under compressive forces. A visualisation of the numerical ratios of jointly and jaw-specific regulated signalling pathways is shown in Fig. 5. Overall, a large proportion of the signalling pathways are only regulated in one jaw (Fig. 5). Detailed information about regulated pathways is shown in additional files 2–5.

Visualization of the a number of jaw-specific signaling pathways that are regulated by mechanical stimulation quantitative relationships between the signaling pathways regulated by mechanical stimulation in the upper and lower jaw, based on various databases: enrichment pathways, Gene Ontology (GO), Kyoto Encyclopedia of Genes and Genomes (KEGG), Wikipathways and Enriched Pathways. (The data shown are derived from experiments performed with PDLSCs obtained from seven different donors.)

## Discussion

Clinical observations suggest that the bone remodeling capacity, with focus on tooth movement rate in the alveolar bone, tends to be faster and more effective in the maxilla compared to the mandible [14–16]. In both upper and lower jaws, PDLSCs are the predominant cell type within the periodontal ligament playing a crucial role in maintaining the periodontal apparatus. These cells contribute to tooth anchorage and nutrition, and are involved in proprioception, force buffering, periodontal remodeling, and the transmission of mechanical stimuli [34]. These properties identify PDLSCs as key regulators of periodontal remodelling [9]. According to current research, the in vitro models available to date provide a comprehensive picture of the processes of periodontal remodelling under mechanical stimulation and its potential modulation possibilities [5, 35–38]. However, none of these studies have distinguished between the maxilla and mandible, meaning that potential jaw-specific intracellular signalling pathways have remained undiscovered, even though clinical observations suggest such differences exist.

These are evident, for example, in increased interleukin levels in the gingival crevicular fluid of the upper jaw, which was obtained from the periodontal ligament surrounding the tooth [23]. 

In this study, we present a novel strategy to investigate jaw-specific metabolic differences in mechanical response and remodeling capacity by analyzing variations in intracellular kinase activity in PDLSCs. This methodology allowed us to discriminate region-specific responses in cells from healthy donors during mechanical stimulation.

By specifically examining PDLSCs from the upper and lower jaw, we were able to show for the first time that the kinase profile after mechanical stimulation differs significantly between the maxilla and mandible. In general, PTKs were mainly downregulated in both jaws while STKs were upregulated. However, the respective number of regulated kinases is striking. While only a few PTKs were downregulated and many STKs were upregulated in the upper jaw, the opposite was true in the lower jaw, with many downregulated PTKs and few upregulated STKs. Interestingly, only some of the affected kinases were equally regulated in the maxilla and mandible. IRR was upregulated in the maxilla and downregulated in the mandible, while Fyn was downregulated in both jaws. The STKs (CDKL2, AMPK[alpha]1, PKG1, and PKA[alpha]) were upregulated in both jaws. All other regulated PTKs and STKs were regulated explicitly in only one jaw. This suggests that, under mechanical stress, only a minority of regulated kinases exhibit a common signalling pathway, while the majority of kinases indicate jaw-specific pathways.

Insulin receptor-related receptor (IRR) is the only kinase that is regulated inversely in both jaws, and first undergoes autophosphorylation. This modification activates its intrinsic tyrosine kinase domain, which in turn triggers the phosphorylation of downstream intracellular targets. By this, IRR facilitates the initiation of two important cellular signaling pathways: the PI3K-AKT axis and the MAPK cascade, both of which are essential for mediating metabolic and growth responses. The activation of IRR by mechanical stimulation is not yet fully understood [39–41]. Fyn, the second PTK which is regulated in both jaws, is known to be involved in regulating cell adhesion, migration, and cytoskeletal dynamics through integrin, FAK, and PI3K/AKT and MAPK signaling [42]. The commonality between Fyn and IRR, a connection to the PI3k-AKT axis and MAPK cascade, suggests they play a relevant role in the cellular response to mechanical stress during orthodontic tooth movement, as it is known that these signalling pathways are regulated under this condition. The same applies to the four STKs regulated in both jaws.

Based on the differences in kinase activity, possible signaling pathways modulated by the mechanical stimulus were reconstructed using the GO, KEGG, Wikipathways, and Enriched Pathways databases. Our analyses revealed distinct relevant signaling pathways for the upper and lower jaws. Interestingly, in addition to numerous signalling pathways that had not previously been associated with mechanical stimulation, the analyses also identified signalling pathways such as PI3K-AKT, MAPK and Wnt, whose relevance for periodontal remodelling had already been demonstrated [5, 9]. 

In summary, our findings validate the hypothesis that PDLSCs from the maxilla and mandible exhibit distinct intrinsic properties, reflected in divergent kinase activity profiles in response to mechanical stimulation. These molecular differences likely contribute to clinically observed variations in tooth movement dynamics and alveolar remodeling capacity. The identification of region-specific kinases provides a valuable entry point for future mechanistic studies and may inform the development of targeted, site-specific therapeutic strategies in orthodontics and periodontal regeneration.

## Conclusion

PDLSCs derived from the upper and lower jaw exhibit distinct intrinsic properties, reflected in differential activation of kinase-driven regulatory pathways under static mechanical stimulation. Recognizing and incorporating these jaw-specific molecular signatures in future research may enhance our understanding of regionally tailored signaling mechanisms, ultimately contributing to more precise approaches for the management of orthodontic tooth movement and periodontal wound healing.

**Table 1:** Detailed information about donors and specific teeth. If two teeth are specified for one jaw, this means that the cells of both teeth have been pooled

Donor Patient	Age (yrs)	Sex	Tooth location (FDI)
1	18	female	18, 28
			38, 48
2	20	female	18, 28
			48
3	31	female	28
			38
4	19	male	28
			38
5	19	male	28
			38, 48
6	18	male	18, 28
			38
7	22	male	18
			48

**Table 2:** A strong difference between the PTK and STK kinase activity profiles was observed when comparing the upper and lower jaws under compressive forces

Upper jaw									
PTK peptides					STK peptides				
ID	Uniprot Accession	Sequence	delta_Force	TTest_p_Force	ID	Uniprot Accession	Sequence	delta_Force	TTest_p_Force
**VGFR3_1061_1073**	**P35916**	**DIYKDPDYVRKGS**	**0**,**535**	**0**,**014**	**RBL2_655_667**	**Q08999**	**GLGRSITSPTTLY**	**0**,**500**	**0**,**003**
**DYR1A_212_224**	**Q13627**	**KHDTEMKYYIVHL**	**0**,**981**	**0**,**032**	**RB_803_815**	**P06400**	**NIYISPLKSPYKI**	**0**,**204**	**0**,**019**
DCX_67_79	O43602	GIVYAVSSDRFRS	-0,172	0,035	**KCNA2_442_454**	**P16389**	**PDLKKSRSASTIS**	**0**,**599**	**0**,**041**
					**FRAP_2443_2455**	**P42345**	**RTRTDSYSAGQSV**	**0**,**614**	**0**,**042**
					**KCNA1_438_450**	**Q09470**	**DSDLSRRSSSTMS**	**0**,**724**	**0**,**045**

Lower jaw									
PTK peptides					STK peptides				
ID	Uniprot Accession	Sequence	delta_Force	TTest_p_Force	ID	Uniprot Accession	Sequence	delta_Force	TTest_p_Force
CDK4_11_23	P11802	EIGVGAYGTVYKA	-0,352	0,002	**CDK7_163_175**	**P50613**	**GSPNRAYTHQVVT**	**0**,**313**	**0**,**005**
EFS_246_258	O43281	GGTDEGIYDVPLL	-0,355	0,008	**RBL2_655_667**	**Q08999**	**GLGRSITSPTTLY**	**0**,**294**	**0**,**024**
YES_420_432	P07947	LIEDNEYTARQGA	-0,306	0,011	**NEK3_158_170**	**P51956**	**FACTYVGTPYYVP**	**0**,**299**	**0**,**045**
EGFR_1103_1115	P00533	GSVQNPVYHNQPL	-1,110	0,012					
CDK2_8_20	P24941	EKIGEGTYGVVYK	-0,244	0,020					
HAVR2_257_267	Q8TDQ0	GIRSEENIYTI	-0,255	0,020					
EPHA2_765_777	P29317	EDDPEATYTTSGG	-0,245	0,022					
PAXI_111_123	P49023	VGEEEHVYSFPNK	-0,238	0,025					
KIT_930_942_C942S	P10721	ESTNHIYSNLANS	-0,203	0,034					
NTRK2_696_708	Q16620	GMSRDVYSTDYYR	-0,260	0,035					
MK12_180_189_M182B	P53778	SEBTGYVVTR	-0,265	0,035					
PECA1_708_718	P16284	DTETVYSEVRK	-0,198	0,036					
PGFRB_768_780	P09619	SSNYMAPYDNYVP	-0,481	0,036					
ENOG_37_49	P09104	SGASTGIYEALEL	-0,283	0,036					
CD79A_181_193	P11912	EYEDENLYEGLNL	-0,270	0,038					
PTN6_531_541	P29350	GQESEYGNITY	-0,212	0,045					

Comparison of significantly different PTK- and STK-specific peptides. Bold text indicates an upregulation by compressive force, regular text indicates a downregulation of peptide phosphorylation. A p-value < 0.05 was considered statistically significant

**Table 3:** Top 10 PTK and STK predicted upstream kinases from lower and upper jaw

Upper jaw							
PTK				STK			
Kinase Name	Kinase Uniprot ID	Mean Kinase Statistic	Median Final Score	Kinase Name	Kinase Uniprot ID	Mean Kinase Statistic	Median Final Score
JAK3	P52333	-0.571	2.996	**CDKL2**	**Q92772**	**0.646**	**3.333**
**IRR**	**P14616**	**0.310**	**1.609**	**ANP alpha**	**P16066**	**0.487**	**2.585**
JAK1 beta	P23458	-0.351	1.552	**CDK11**	**Q9BWU1**	**0.576**	**2.465**
EphB3	P54753	-0.373	1.345	**HGK/ZC1**	**O95819**	**0.647**	**2.403**
Fyn	P06241	-0.221	1.266	**CDK4**	**P11802**	**0.542**	**2.303**
				**CDK6**	**Q00534**	**0.537**	**2.303**
				**MAPKAPK3**	**Q16644**	**0.442**	**2.179**
				**SGK2**	**Q9HBY8**	**0.506**	**2.049**
				**CDKL5**	**O76039**	**0.438**	**1.917**
				**ATR**	**Q13535**	**0.530**	**1.892**

Lower jaw							
PTK				STK			
Kinase Name	Kinase Uniprot ID	Mean Kinase Statistic	Median Final Score	Kinase Name	Kinase Uniprot ID	Mean Kinase Statistic	Median Final Score
Lyn	P07948	-0.826	3.690	**JNK2**	**P45984**	**0.297**	**1.893**
SyK	P43405	-0.646	3.665	**PKG1**	**Q13976**	**0.251**	**1.610**
ZAP70	P43403	-0.662	3.627	**CDKL2**	**Q92772**	**0.331**	**1.563**
EphA5	P54756	-1.393	2.887	**CDK2**	**P24941**	**0.277**	**1.494**
TRKB	Q16620	-0.686	2.788	**AMPK alpha**	**Q13131**	**0.301**	**1.419**
Abl	P00519	-0.637	2.695	**PKN1/PRK1**	**Q16512**	**0.303**	**1.390**
Arg	P42684	-0.647	2.600	**PKA alpha**	**P17612**	**0.232**	**1.354**
EphA8	P29322	-1.112	2.473	**JNK3**	**P53779**	**0.256**	**1.236**
Fgr	P09769	-0.978	2.414	**JNK1**	**P45983**	**0.252**	**1.215**
Src	P12931	-0.678	2.370	**CDC2/CDK1**	**P06493**	**0.241**	**1**,**213**

Bold text indicates an upregulation by compressive force, regular text indicates a downregulation

**Table 4:** Pathway prediction

Regulated only in lower jaw			Regulated in both jaws			Regulated only in upper jaw		
**Imflammation**								
ID	Description	KinaseRatio	ID	Description	KinaseRatio	ID	Description	KinaseRatio
R-HSA-2,682,334	EPH-Ephrin signaling	12/47	R-HSA-3,928,664	Ephrin signaling	3/47	R-HSA-450,302	activated TAK1 mediates p38 MAPK activation	3/33
R-HSA-450,321	JNK (c-Jun kinases) phosphorylation and activation	3/47	hsa04657	IL-17 signaling pathway	3/44	R-HSA-389,357	CD28 dependent PI3K/Akt signaling	2/33
R-HSA-199,418	Negative regulation of the PI3K/AKT network	10/47	WP286	IL-3 signaling pathway	7/47	GO:0071353	cellular response to interleukin-4	2/39
R-HSA-6,811,558	PI5P, PP2A and IER3 Regulate PI3K/AKT Signaling	10/47	hsa04750	Inflammatory mediator regulation of TRP channels	6/44	GO:0019221	cytokine-mediated signaling pathway	6/39
GO:0043551	regulation of phosphatidylinositol 3-kinase activity	7/52	R-HSA-448,424	Interleukin-17 signaling	3/47	R-HSA-198,753	ERK/MAPK targets	2/33
			R-HSA-450,294	MAP kinase activation	3/47	WP195	IL-1 signaling pathway	4/35
			R-HSA-5,683,057	MAPK family signaling cascades	12/47	WP49	IL-2 signaling pathway	5/35
			WP382	MAPK signaling pathway	8/47	WP395	IL-4 signaling pathway	5/35
			hsa04010	MAPK signaling pathway	16/44	WP127	IL-5 signaling pathway	3/35
			R-HSA-5,684,996	MAPK1/MAPK3 signaling	11/47	WP205	IL-7 signaling pathway	3/35
			R-HSA-166,166	MyD88-independent TLR4 cascade	3/47	R-HSA-8,983,432	Interleukin-15 signaling	2/33
			WP4172	PI3K-Akt signaling pathway	14/47	R-HSA-451,927	Interleukin-2 family signaling	2/33
			hsa04151	PI3K-Akt signaling pathway	17/44	R-HSA-9,020,558	Interleukin-2 signaling	2/33
			R-HSA-1,257,604	PIP3 activates AKT signaling	10/47	R-HSA-8,854,691	Interleukin-20 family signaling	2/33
			R-HSA-5,673,001	RAF/MAP kinase cascade	10/47	R-HSA-9,020,958	Interleukin-21 signaling	2/33
			R-HSA-449,147	Signaling by Interleukins	10/47	R-HSA-1,266,695	Interleukin-7 signaling	2/33
			R-HSA-187,687	Signalling to ERKs	2/47	hsa04630	JAK-STAT signaling pathway	4/33
			hsa04668	TNF signaling pathway	3/44	R-HSA-5,674,135	MAP2K and MAPK activation	3/33
			R-HSA-168,142	Toll Like Receptor 10 (TLR10) Cascade	3/47	R-HSA-5,684,264	MAP3K8 (TPL2)-dependent MAPK1/3 activation	2/33
			R-HSA-181,438	Toll Like Receptor 2 (TLR2) Cascade	3/47	WP422	MAPK cascade	4/35
			R-HSA-168,164	Toll Like Receptor 3 (TLR3) Cascade	3/47	R-HSA-5,675,221	Negative regulation of MAPK pathway	3/33
			R-HSA-168,176	Toll Like Receptor 5 (TLR5) Cascade	3/47	R-HSA-171,007	p38MAPK events	3/33
			R-HSA-168,181	Toll Like Receptor 7/8 (TLR7/8) Cascade	3/47	GO:0007259	receptor signaling pathway via JAK-STAT	4/39
			R-HSA-168,138	Toll Like Receptor 9 (TLR9) Cascade	3/47	GO:0070670	response to interleukin-4	2/39
			R-HSA-168,179	Toll Like Receptor TLR1:TLR2 Cascade	3/47	WP3851	TLR4 signaling and tolerance	3/35
			R-HSA-168,188	Toll Like Receptor TLR6:TLR2 Cascade	3/47	R-HSA-75,893	TNF signaling	4/33
			hsa04620	Toll-like receptor signaling pathway	3/44	R-HSA-166,016	Toll Like Receptor 4 (TLR4) Cascade	7/33
			R-HSA-937,061	TRIF(TICAM1)-mediated TLR4 signaling	3/47	R-HSA-168,898	Toll-like Receptor Cascades	7/33
						WP75	Toll-like receptor signaling pathway	4/35
						WP3858	Toll-like receptor signaling related to MyD88	3/35
						R-HSA-933,542	TRAF6 mediated NF-kB activation	2/33
						WP4482	Vitamin D in inflammatory diseases	3/35
**Bone remodeling**								
GO:0098751	bone cell development	2/52	WP673	ErbB signaling pathway	11/47	R-HSA-3,858,494	Beta-catenin independent WNT signaling	3/33
GO:0198738	cell-cell signaling by wnt	5/52	hsa04012	ErbB signaling pathway	11/44	WP1434	Osteopontin signaling	2/35
GO:0001503	ossification	5/52	hsa04380	Osteoclast differentiation	6/44	R-HSA-195,721	Signaling by WNT	4/33
GO:0033687	osteoblast proliferation	2/52	WP2018	RANKL/RANK signaling pathway	6/47	WP363	Wnt signaling pathway	3/35
GO:0030177	positive regulation of Wnt signaling pathway	4/52	hsa04310	Wnt signaling pathway	4/44			
GO:0046850	regulation of bone remodeling	2/52						
GO:0045124	regulation of bone resorption	2/52						
R-HSA-8,941,326	RUNX2 regulates bone development	3/47						
R-HSA-8,940,973	RUNX2 regulates osteoblast differentiation	3/47						
R-HSA-1,227,986	Signaling by ERBB2	7/47						
**Mechanical/external Stimulation**								
GO:0042490	mechanoreceptor differentiation	2/52	GO:0032103	positive regulation of response to external stimulus	11/52			
WP4534	Mechanoregulation and pathology of YAP/TAZ	4/47						
GO:0050954	sensory perception of mechanical stimulus	3/52						
**Vascularisation**								
hsa04015	Rap1 signaling pathway	8/44	R-HSA-194,138	Signaling by VEGF	5/47	hsa04370	VEGF signaling pathway	4/33
			R-HSA-4,420,097	VEGFA-VEGFR2 Pathway	5/47			
			WP3888	VEGFA-VEGFR2 signaling pathway	10/47			

Selected predicted pathways from the databases GO (GO: ), KEGG (hsa), Wikipathways (WP) and enriched pathways (R-HAS) with relevance for the biological processes of periodontal remodeling

## Supplementary Information

Supplementary Material 1

Supplementary Material 2

## Data Availability

The datasets supporting the conclusions of this article are included within the article and its additional files.
